# Bird Eye View of Protein Subcellular Localization Prediction

**DOI:** 10.3390/life10120347

**Published:** 2020-12-14

**Authors:** Ravindra Kumar, Sandeep Kumar Dhanda

**Affiliations:** 1 Biometric Research Program, Division of Cancer Treatment and Diagnosis, National Cancer Institute, NIH, 9609 Medical Center Drive, Rockville, MD 20850, USA; 2 Department of Oncology, St. Jude Children’s Research Hospital, Memphis, TN 38105, USA

**Keywords:** protein localization, signal peptide-based method, sequence compositional information-based method, machine learning based method, integrated method

## Abstract

Proteins are made up of long chain of amino acids that perform a variety of functions in different organisms. The activity of the proteins is determined by the nucleotide sequence of their genes and by its 3D structure. In addition, it is essential for proteins to be destined to their specific locations or compartments to perform their structure and functions. The challenge of computational prediction of subcellular localization of proteins is addressed in various in silico methods. In this review, we reviewed the progress in this field and offered a bird eye view consisting of a comprehensive listing of tools, types of input features explored, machine learning approaches employed, and evaluation matrices applied. We hope the review will be useful for the researchers working in the field of protein localization predictions.

## 1. Introduction

**Figure 1:**
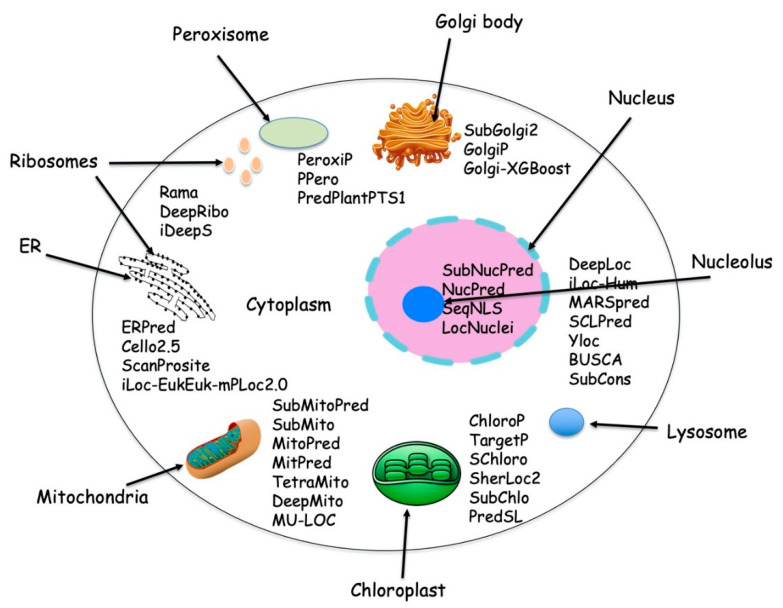
Typical cell with different subcellular location and with available protein localization prediction tools.

Proteins are localized into different cellular compartments and sub-compartments inside the cell. Each subcellular compartment has a distinct well-defined function in the cell and has a characteristic physicochemical environment, which drives proper functioning of the proteins. Each subcellular compartment has a distinct, well defined function in the cell and is considered to have evolved from the prokaryotic cell. Typical eukaryotic cells have two types of DNAs (i) chromosomal nuclear DNA and (ii) organelle DNA, which is present in mitochondria and chloroplast while prokaryotic cells have only single type of DNA called nucleoid. The nuclear DNA encodes the majority of proteins while only a small number of proteins are encoded by organelle DNA. Eukaryotic cells can synthesize up to 100,000 different types of protein [1], which are destined for one or more predetermined subcellular locations. Figure 1 depicts various protein localization prediction methods available for different cellular compartments.

**Figure 2:**
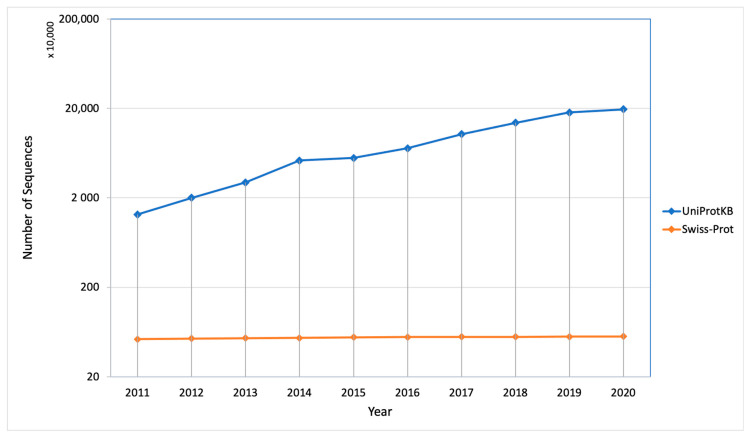
Number of sequences deposited and manually annotated proteins in the UniProt database in the last 10 years.

The protein synthesis occurs in the cytoplasm and then the newly synthesized proteins are further transported to their destined compartment to execute their function. Protein must be targeted to the right compartment in cells to perform their function and mis-localization of the proteins leads to functional loss or disorder, which contributes to many human diseases including cardiovascular, neurodegenerative disease and cancers [2,3]. Assigning subcellular localization for protein is a significant step to elucidate its interaction partners and predict their functions or potential roles in the cellular machinery [4]. There are a number of sequences that are deposited every year in the UniProt Knowledgebase (UniProtKB) but only a few of them were manually annotated and reviewed (UniProtKB/SwissProt), which explains the gap between the deposited sequence and annotated sequence is increasing every year (Figure 2). Therefore, there is a need of computational methods to predict subcellular localization with high quality and accuracy, which is of great significance in understanding cellular proteome and also helpful in designing the drug or targets. To date, many efforts have been made in this regard. Based on different kinds of characteristics, several machine learning approaches have been developed such as neural networks [5,6], hidden Markov models [7,8,9], support vector machines [10,11,12], deep learning [13,14,15], random forest [16], and extreme gradient boosting [17] for prediction of subcellular localization of proteins.

The purpose of this review is not only to make a complete list of protein localization prediction methods but also describe those methods that imply significant developments in this field. In this review we primarily focus on the eukaryotic prediction method however, some of the mentioned subcellular localization prediction methods could be used for both the prokaryotic and eukaryotic cell. This review focuses on (1) available computational methods for subcellular localization prediction, (2) different algorithms used in the development of these methods, (3) various features used in prediction purposes, and (4) future aspect and importance of subcellular localization in the biological field.

## 2. Experimental Approaches for Protein Localization

Several experimental methods are available for determining protein localization, but the most common method is to label the protein of interest with fluorescent probes and then visualize the distribution of the protein within cells under a fluorescence microscope such as immunofluorescence microscopy, immunolocalization, mass spectrometry, co-expression of fluorescent proteins, and electron microscopy. Fractionation based approach such as gradient centrifugation and 2D gel electrophoresis are also a widely used method to experimentally establish the localization of a protein. These experimental methods are relatively expensive and time consuming, which explains for a large information gap existing between known protein and their location information. Consequently, various computational methods have been developed to help fill this void. In this review, we focused only on the computational approaches and tools for prediction of protein localization.

## 3. Computational Approaches for Protein Localization

With the rapid development of advanced genome sequencing methods, the complete genome sequences are increasing day by day and the challenges for computational biologists are to manage, analyze, and annotate this plethora of unprocessed raw biological data. To now, a number of computational methods have been developed to solve this problem. While many have attempted to explore uncharacterized protein information, others have used the whole proteome sequence information to develop new machine learning algorithms for different things such as the prediction of motifs, prediction of ligand binding sites, etc. Based on protein sequence information, the computational method can be divided into the following categories: (1) sequence feature-based methods, (2) homology-based methods, (3) protein domain and motif information-based methods, (4) signal peptide-based methods, (5) non-sequence derived features-based methods, and (6) integrated methods, which could use a combination of two or more methods.

### 3.1. Sequence Feature-Based Method

Sequence features are commonly used in localization prediction since some differences in the sequence features are empirically known to be correlated with different localization sites. Nishikawa and Ooi [18] first noted the correlation of amino acid composition to its biological and functional character in 1982. After that in 1983 Nakashima developed the first sequence-based method for subcellular localization [19]. They used amino acid composition to discriminate between intracellular and extracellular proteins. Later several research groups successfully used amino acid composition as a tool for subcellular localization predictions [16,20,21].

In sequence feature-based methods, the complete sequence of proteins is transformed into a numerical feature vector, which is then used to predict the subcellular location. There are different types of sequence feature-based methods available: (i) amino acid composition based method, in which the frequency of 20 different amino acids is calculated but it ignores the sequence order information of each residue. (ii) Chou’s Pseudo amino acid composition (PseAAC) [22], which considers the amino acid composition along with the potential interaction among the adjacent residues. This can be further categorized into different modes of PseAAC such as the gene ontology mode, functional domain mode and sequential evolution mode. (iii) Hybrid method, which allows the integration of different parameters or features for the prediction and usually results in an increased the prediction performance [4].

### 3.2. Homology Based Method

This is the most common way to predict the uncharacterized protein on the basis of the presence of homologous sequences of known function with an assumption that function is evolutionarily conserved [23]. This approach first identifies for homologous sequences in the proteins with known subcellular location and then extrapolates to predict the location of unknown proteins, hence this approach is also known as “Annotation by Homology Transfer”. Homology is a qualitative term, which attributes evolutionary relationships among different protein sequences. Orthologous proteins also typically have similar sequences and thus similar subcellular localization patterns. Proteins with a highly similar sequence correlate well with the cellular localization site while those with dissimilar sequences indicate that they are distant and may or may not be colocalized. In 2002, Nair and Rost [24] showed the correlation between sequence similarity with subcellular localization. They considered 11 different compartments and observed sequence conservation among the major compartments. BLAST, PSI-BLAST, and hidden Markov models (HMM) are routinely used for searching homologous sequences. The limitation of homology-based methods is more pronounced in cases where no homology is found between the query sequence and the annotated proteins sequence. Additionally, it is known that a single amino acid substitution in localization signals can change the localization of a protein [25,26,27]. Thus, sequence homology is a noncausal feature for the localization prediction and should be used with caution when applied to nonnative sequences or in case when homology is less [28].

### 3.3. Functional Motifs, Domains, and other Signatures Based Method

Proteins have evolved in different compartments, which limit their interactions with other proteins and ultimately impact their functions. Some of these proteins preserved some sequential or structural patterns or motifs. Though not all of these motifs and domains are specific to subcellular localization, many preferentially occur in some specific compartments and such domains can be used to predict the localization of any proteins. Studying proteins at a domain/motif level allows more accurate functional inference [29]. In 2002, Mott et al. [30] first used 300 Simple Modular Architecture Research Tool (SMART) domains to predict three subcellular locations viz secreted, cytoplasm, and nucleus. After that, several works have been used for the protein motif and domains as features for protein localization predictions [31,32].

These motifs are not just limited to sequence patterns, but also extended to the structural information. There are a couple of tools such as PROSITE [33] and MEME [34] that employed this feature to use for protein localization. While the structure is not available for a big chunk of protein sequences, this gap is filled by several proteins structure predictions servers, like I-TASSER and C-I-TASSER servers [35]

### 3.4. Signal Peptide Based Method

Signal peptides are short amino acid sequences in the amino terminus of the newly synthesized proteins and are found in all organisms including bacteria, archaea, and eukaryotes. The function of the signal peptide is to enable the transport machinery to translocate the proteins to different subcellular locations. They are present in secretory proteins and in transmembrane proteins and the protein residing in different eukaryotic organelles have different types of signal peptide sequences [36]. The signal peptide is followed by a stretch of amino acids that form the cleavage site recognized by peptidases and the signal peptide is removed after translocation, except in the case of transmembrane proteins. In case of transmembrane proteins, this signal peptide serves as signal anchor sequences. The importance of various signal peptide sequences in proteins in their subcellular localization has led to attempts to predict the subcellular location on the basis of the signal peptide present in proteins. The prediction of the signal peptide involves two main tasks: (1) discriminating between the signal peptide and signal anchor sequences and (2) also predicting the position of the signal peptide cleavage site [37]. The major challenge in signal peptide prediction is discriminating between true signal sequences and other hydrophobic regions. In addition to it, the accurate prediction of the cleavage site is also very important due to the high variability of the signal sequence length and the absence of sequence motifs that unambiguously mark the position of the cutting site [38]. A number of prediction methods are available that recognize and predict the subcellular location on the basis of signal peptides (Table 1). SignalP was the first publicly available method [15] and there are many versions available, which were developed based on different methods. Version-1 [39] was based on artificial neural networks, while version-2 [40] was based on hidden Markow models, version-3 [41] has an improved cleavage site prediction, version-4 [42] has improved discrimination of signal peptides and TM helices, and version-5 [38] is a deep neural network-based method combined with a conditional random field classification and an optimized transfer learning for improved signal peptide prediction.

The signal peptide-based method is a good approach to predict the proteins that contain the signal peptide, but it has some drawbacks, which make these methods not able to be applied for proteome scale prediction. (i) Not all proteins contain signal peptides. There are many proteins that do not have any reported signal peptide sequence and despite this are still translocated to their respective subcellular location. (ii) Many proteins follow the “piggyback import” mechanism during protein translocation, which means these proteins do not have any specific signal peptide for the localization, but they interact and bind to different proteins that have a signal peptide for translocation and then are co-imported to specific target locations [43,44].

### 3.5. Non-Sequence Derived Features

A variety of non-sequence derived features have been used to predict subcellular localization. For example, LOC3D [45], which used the structural information for identification and prediction of proteins subcellular locations. There are a number of non-sequence derived features that have been used in an automated classifiers including immunohistochemistry [3,46,47], fluorescence microscopy image [48,49], protein–protein interaction (PPI) data [50], expression data [51], and recommendation systems [52].

### 3.6. Integrated Method

The different strategies for predicting protein localization have their own strengths and weaknesses. To enhance the performance of prediction methods, it is important to combine multi-characteristic strategies, which give more complete information to understand the relationship between protein localization with its sequence, structure, physicochemical properties, and function. Hence a combination of different input vectors and different tools will be the successful strategy in protein subcellular localization prediction. Many methods have successfully utilized the combination of protein features to enhance the performance of protein subcellular localization predictions. The Protein Subcellular Localization Prediction Tool (PSORT) family method is one of the integrated methods, which contains several tools for localization prediction. The family includes a number of tools: (i) PSORT [53], the first integrated method of the PSORT family (http://psort.org) for the plant and bacterial protein, (ii) PSORT II [54] for yeast and animal proteins, (iii) iPSORT [55] for N-terminal sorting signals for plant or non-plants; (iv) PSORTb [56,57,58] for bacterial and archaeal proteins, and (v) WoLF PSORT [59] for eukaryotic proteins including plants, animals, and fungi.

A similar approach was taken by many researchers where they integrated biological or empirical sequence features correlated with subcellular location with a variety of machine-learning algorithm such as KNN, SVM, and deep learning: MultiLoc, integration of the phylogenetic profile and GO terms of retrieved homologues such as MultiLoc2, CELLO2.5, SherLoc2, YLoc, iLoc-Euk, Loctree3, DeepLoc, etc. People are also integrating different computational tools for predicting subcellular localization. The Bologna Unified Subcellular Component Annotator (BUSCA) [60] is an example of such an integrated tool where the author combines methods for identifying signal and transit peptides (DeepSig and TPpred3), GPI-anchoes (PredGPI), and transmembrane domains (ENSEMBLE3.0 and BetAware) with tools for discriminating subcellular localization of both globular and membrane proteins (BaCelLo, MemLoci, and SChloro). This integrated method performs better than the other methods based on single feature approaches. There are a number of recently developed subcellular localization methods available, which are used by a wide range of researchers (Table 2 and Table S1).

## 4. Machine Learning Tools Used in Protein Prediction

**Figure 3:**
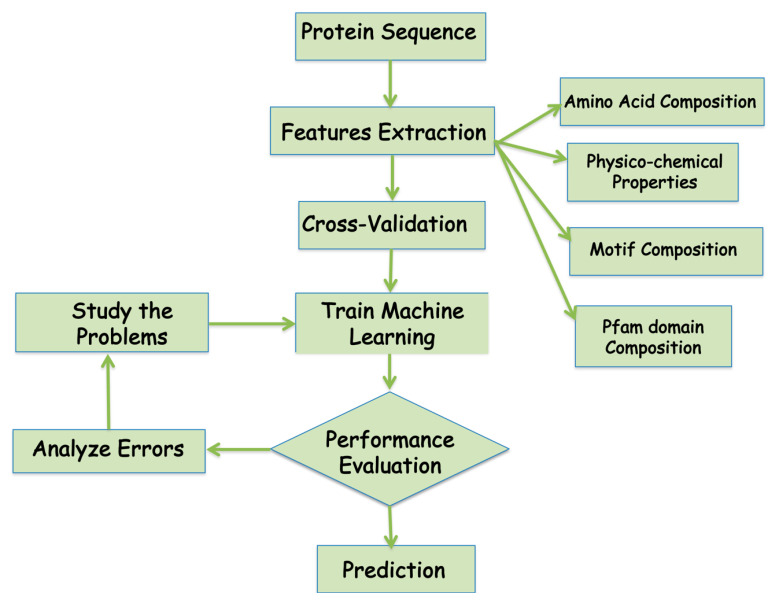
Flowchart of the development of the machine learning algorithm for the prediction of protein localization.

A number of machine learning methods have been successfully employed for protein subcellular localization predictions using the sequence information for all the proteins with known subcellular localization information to learn and develop a meaningful representation or representative model of the biological data. In the machine learning, the biological data is first categorized into classes based on the Figure 3, which represents the flowchart of a typical machine learning approach for subcellular protein localization. The performance of machine learning model has to be tested using various parameters. Below are a few parameters that are widely used to analyze machine learning performances (Table 3).

### 4.1. Support Vector Machine

Support vector machine (SVM) is a supervised machine learning tool, which is used for classification and regression purposes for complex biological problems. It is based on statistical learning theory and was developed by Vapnik in the year 1995 [65]. It can solve linear and non-linear issues well for many problems such as subcellular localization prediction, different protein family classification prediction. The objective of an SVM is to find a hyperplane in a N-dimensional space that classifies the data into different classes with maximum margin. There are a number of protein subcellular localization methods available that used SVM as a prediction tool [7,11,21,66]. For implementation of SVM, people used different packages of SVM like SMV_light, LIBSVM, Caret in R, and Scikit-Learn for python.

### 4.2. Random Forest

Random forest (RF) is an ensemble learning method for classification and regression analysis. The key element of RF is to build multiple decision trees and merge them together to get a more accurate and stable prediction. This is the most popular choice for bioinformaticians to analyze the complex biological data. Apart from subcellular localization prediction [2], RF is applied in a variety of problems such as gene expression classification [67,68], biomarker discovery [69,70,71,72], protein–protein interaction [73,74], and cancer drug predictions [75,76,77,78].

### 4.3. Neural Network and Deep Learning

The successes of neural networks have led to the development of various programming frameworks to build and train neural network models. The traditional neural network architecture is the feed forward neural network with one hidden layer, in which each input neuron is connected to each neuron in the hidden layer and which is further connected to each neuron in the output layer. Apart from the subcellular localization prediction [13,79], deep learning is successfully applied in many other biological fields such as the prediction of splicing pattern prediction [80,81], protein secondary structure prediction [82,83], different types of cancer and drug-target interactions [84,85,86,87], and the patterns in the biomedical imaging datasets [88].

There are some popular python libraries, which implements deep learning such as PyTorch, Caffe, TensorFlow, Theano, Keras, and Lasagne [89]. Keras is the most powerful and easy to use python library for developing and evaluating deep learning models.

## 5. Importance and Future Aspects of Protein Subcellular Localization

Protein subcellular localization is getting more attention as the function of the protein requires its localization to a destined compartment. With increase in genomics and proteomics sequencing has led to the accumulation of a large number of sequences in different databases. We believe that the computational methods developed using the integration of different sources of information will be the key factor to resolve these issues. Most of the current prediction methods are feature based methods, which used sequence information or physicochemical properties. The drawback of these feature-based methods is that it does not utilize rich network information of the proteins such as the gene co-expression network, genetic interaction, and metabolic network [90,91]. There are few methods available that also use PPI [92] and the metabolic network [93] for protein localization predictions. The proteins interacting with each other tend to localize within the same subcellular compartments and thus, the PPI information could be useful in predicting subcellular localizations [25]. Additionally, it is important to explore more interacting partners information to develop a more accurate localization prediction method. Another thing that we need to improve in development of protein localization prediction methods is the basis of most of the methods is that a particular protein can be destined to a single subcellular location, which lacks the capability to predict the proteins present in multiple locations. More efforts are needed for development of protein localization methods to address proteins present in multiple locations. There is still some room for major improvements in the multicellular localization prediction with high reliability while in many ways the field still holds interesting challenges for the bioinformatician.

## Figures and Tables

**Table 1:** Some useful signal peptide-based methods.

Method	Tools Used	Performance Matrix	Locations/Organism	Availability	Year
SignalP-5.0 *	convolutional and recurrent (LSTM) neural networks	MCC, precision and recall	Archaea, Gram-positive Bacteria, Gram-negative Bacteria and Eukarya	http://www.cbs.dtu.dk/services/SignalP/	2019
TargetP 2.0 *	recurrent neural networks (RNNs) network	Precision, recall, F1-score, MCC	mitochondrial, chloroplastic, secretory pathway	http://www.cbs.dtu.dk/services/TargetP/	2019
SigUNet	Convolutional neural network	MCC, precision, recall, F1 measure	Eukaryotes, Gram-positive and Gram-negative bacteria	https://github.com/mbilab/SigUNet	2019
DeepSig	Convolutional Neural Networks	MCC, False Positive Rate, precision and recall	Eukaryotes, Gram-positive bacteria and Gram-negative bacteria	https://deepsig.biocomp.unibo.it	2018
SChloro	SVM	Accuracy, Recall, Precision, F1-score, and MCC	six chloroplastic sub-compartments	http://schloro.biocomp.unibo.it	2017
PredSL	combination of neural networks, Markov chains, scoring matrices (PrediSi), and HMMs,	Accuracy	Eukaryotic subcellular location	http://bioinformatics.biol.uoa.gr/PredSL/	2006
TatP	HMM/artificial neural networks.	S-score and the C-score, Y-score, D-score	bacteria	http://www.cbs.dtu.dk/services/TatP/	2005
ChloroP	Neural network	MCC, sensitivity, specificity	chloroplast transit peptides	http://www.cbs.dtu.dk/services/ChloroP/	1999

* There are different versions of the software available, but here we mentioned only the recent one.

**Table 2:** List of subcellular localization methods.

Method	Tools Used	Performance Matrix	Feature Based	Locations/Organism	Availability	Year
DeepPred-SubMito	Convolutional neural network	Accuracy, MCC	Sequence information	Mitochondrial and submitochondrial proteins	https://github.com/jinyinping/DeepPred-SubMito.git	2020
SubMito-XGBoost	Extreme gradient boosting (XGBoost)	Sensitivity, Specificity, False positive rate, MCC, F1-measure, precision	Sequence information	Submitochondrial proteins	https://github.com/QUST-AIBBDRC/SubMito-XGBoost/	2020
mRNALoc	SVM	Sensitivity, Specificity, Accuracy, MCC	Sequence information	eukaryotic	http://proteininformatics.org/mkumar/mrnaloc	2020
SCLpred-EMS	Convolutional neural network	Sensitivity, Specificity, False positive rate, MCC	Sequence information	endomembrane system and secretory pathway	http://distilldeep.ucd.ie/SCLpred2/	2020
BUSCA	Integrated method of DeepSig, TPpred3, PredGPI, BetAware and ENSEMBLE3.0	Precision, recall, F1-score, MCC	Sequence information, signal and transit peptides, glycophosphatidylinositol (GPI) anchors and transmembrane domains	Gram-positive, gram-negative, fungi, plant, animal	http://busca.biocomp.unibo.it	2018
SubMitoPred	SVM	Sensitivity, Specificity, Accuracy, MCC	Sequence and domain information	Mitochondrial and submitochondrial proteins	http://proteininformatics.org/mkumar/submitopred/	2018
pLoc-mEuk	ML-GKR (multi-label Gaussian kernel regression) classifier	Coverage, Accuracy, Absolute true, Absolute false	Gene Ontology and Chou’s general PseAAC	22 different subcellular localizations of eukaryotic proteins	http://www.jci-bioinfo.cn/pLoc-mEuk/	2018
ERPred	SVM	Sensitivity, Specificity, Accuracy, MCC	Sequence information,	ER Proteins	http://proteininformatics.org/mkumar/erpred/index.html	2017
DeepLoc	deep recurrent neural networks	Accuracy, MCC	Sequence information	10 different location of eukaryotic proteins	http://www.cbs.dtu.dk/services/DeepLoc	2017
SubNucPred	SVM	Sensitivity, Specificity, Accuracy, MCC	Sequence and domain information	Nuclear and subnuclear protein	http://proteininformatics.org/mkumar/subnucpred/	2014
LocTree3	SVM and homology	Accuracy, recall, standard deviation, standard error	Homology-based, Gene Ontology	18 classes for eukaryotes, in six for bacteria and in three for archaea	http://www.rostlab.org/services/loctree3	2014
PlantLoc	localization motif search	accuracy	localization motif information	11 different location of plant proteins	http://cal.tongji.edu.cn/PlantLoc/	2013
iLoc-Cell, package of predictors for subcellular locations of proteins. It includes iLoc-Hum, iLoc-Animal, iLoc-Plant, iLoc-Euk, iLoc-Virus, iLoc-Gpos, iLoc-Gneg	multi-label learning, multi-label KNN	Accuracy, Precision, Recall, Absolute-true rate, Absolute-false rate,	Sequence information, gene ontology, PSSM,	Different subcellular location of Human, animals, plants, eukaryotic, Virus, gram-positive, gram-negatives	http://www.jci-bioinfo.cn/iLoc-Cell	2011, 2012, 2013
MARSpred	SVM	Sensitivity, specificity, Accuracy, MCC	Sequence information, PSSM	cytosolic and mitochondrial aminoacyl tRNA synthetase	http://www.imtech.res.in/raghava/marspred/	2012
SCLPred	Neural Network	Sensitivity, specificity, False positive rate, MCC	primary sequence and multiple sequence alignments	four classes for animals and fungi and five classes for plants	http://distill.ucd.ie/distill/	2011
AtSubP	SVM	Sensitivity, specificity, error rate, MCC, ROC curve	Sequence information, PSSM	subcellular localization of Arabidopsis	http://bioinfo3.noble.org/AtSubP	2010
Euk-mPLoc 2.0	OET-KNN (Optimized Evidence-Theoretic K-Nearest Neighbor) classifiers	accuracy	gene ontology information, functional domain information, and sequential evolutionary information	eukaryotic proteins among the following 22 locations	http://www.csbio.sjtu.edu.cn/bioinf/euk-multi-2/	2010
PSORTb	SVM	Precision, recall, accuracy, MCC	Sequence information	Different subcellular location of Gram-negative, Gram-positive, archaea	http://www.psort.org/psortb	2010
YLoc	naïve Bayes alongside entropy-based discretization	overall accuracy, F1-score	Sequences information, GO-term and motif	animal, fungal and plant proteins	www.multiloc.org/YLoc	2010
SubChlo	evidence-theoretic *K*-nearest neighbor (ET-KNN) algorithm	overall accuracy, accuracy	Sequences information (PseAAC),	chloroplast proteins	http://bioinfo.au.tsinghua.edu.cn/subchlo	2009
MultiLoc2	SVM	Sensitivity, specificity, Accuracy, MCC	phylogenetic profiles and gene ontology terms	Plant, Animal, Fungal	https://abi-services.informatik.uni-tuebingen.de/multiloc2/webloc.cgi	2009
AAIndexLoc	SVM	Sensitivity, specificity, Accuracy, MCC	Sequence information and physicochemical properties	Animal, Fungal and plants	http://aaindexloc.bii.a-star.edu.sg	2008
Cell-PLoc package of predictors for subcellular locations of proteins. It includes Euk-mPLoc, Hum-mPLoc, Plant-PLoc, Gpos-PLoc, Gneg-PLoc, Virus-PLoc	KNN or OET-KN algorithm	Accuracy and F1 score	GO and functional domain information	22 subcellular location of eukaryotic, human, plant, Gram-positive bacterial, Gram-negative bacterial and viral proteins	http://chou.med.harvard.edu/bioinf/Cell-PLoc	2008
ProLoc-GO	SVM-GO, k-NN-GO and fuzzy k-NN-GO	MCC	GO term information	eukaryotic, human,	http://iclab.life.nctu.edu.tw/prolocgo	2008
ProLoc	SVM	Accuracy	physicochemical composition	subnuclear localizations	http://iclab.life.nctu.edu.tw/proloc	2007
SherLoc	SVM	Sensitivity, specificity, MCC	Sequence information	eukaryotic proteins	http://www-bs.informatik.uni-tuebingen.de/Services/SherLoc/	2007
MitPred	SVM	Sensitivity, specificity, Accuracy, MCC	Sequence information	Mitochondrial proteins	http://www.imtech.res.in/raghava/mitpred/	2006
BaCelLo	SVM	Coverage, Normalized Accuracy, geometric average , overall accuracy, Generalized Correlation	Sequence information	Plant, Animal, Fungal	http://www.biocomp.unibo.it/bacell/	2006
HSLpred	SVM	Accuracy, MCC, Reliability index	Sequence information	Human Protein	http://www.imtech.res.in/raghava/hslpred/	2005
PSLpred	SVM	Accuracy, MCC, Reliability index	Sequence information	gram-negative bacterial proteins	http://www.imtech.res.in/raghava/pslpred/	2005
ESLpred	SVM	Accuracy, MCC, Reliability index	Sequence information and PSSM	eukaryotic proteins	http://www.imtech.res.in/raghava/eslpred/	2004

**Table 3:** List of commonly used metrics found in protein subcellular localization prediction.

Metric	Explanation	Formula	References
Accuracy	It is the ratio of the number of correct predictions to the total number of input samples. It works well if there are an equal number of samples belonging to each class.	TP + TN/(TP + FP + TN + FN)	[61,62]
Specificity/True Negative Rate	Proportion of negatives that are correctly identified	TN/(TN + FP)	[61,62]
Precision/Positive Predictive Value	It tells us how many of the correctly predicted cases actually turned out to be positive.	TP/(TP + FP)	[61]
Recall/Sensitivity/True Positive Rate	It tells us how many of the actual positive cases we were able to predict correctly with our model.	TP/(TP + FN)	[61,62]
F1-Score	F1 score is the harmonic mean between precision and recall. Greater the F1 score, better the performance of the model. It tells how precise the classifier is and how robust it is.	F1 = 2 * precision * Recall/Precision + Recall	[63]
Negative Predictive Value	It is defined as the proportion of predicted negatives, which are real negatives. It reflects the probability that a predicted negative is a true negative.	TN/(TN + FN)	[64]
False Positive Rate	Number of incorrect positive predictions divided by the total number of negatives.	FP/(FP + TN)	[61]

Note: TP, TN, FP, and FN represent true positive, true negative, false positive, and false negative respectively.

## Supplementary Materials

The following are available online at https://www.mdpi.com/2075-1729/10/12/347/s1, Table S1: Comparative performance of available subcellular localization methods.
