# Supplementary material for: Bird Eye View of Protein Subcellular Localization Prediction
Source: Life (Basel). 2020 Dec 14;10(12):347. doi: 10.3390/life10120347 (PMC7764902; doi:10.3390/life10120347)
Supplement: Supplementary file 1 [file life-10-00347-s001.pdf]

Review

Bird Eye View of Protein Subcellular Localization Prediction

Ravindra Kumar [1,*] and Sandeep Kumar Dhanda [2,*]

[1] Biometric Research Program, Division of Cancer Treatment and Diagnosis, National Cancer Institute, NIH, 9609 Medical Center Dr., Rockville, MD 20850, USA

[2] Department of Oncology, St. Jude Children's Research Hospital, Memphis, TN 38105, USA

* Correspondence: rbanaudha@gmail.com (R.K.); sandeep.dhanda@stjude.org (S.K.D.)

Table 1. Comparative performance of available subcellular localization methods.

Method	Sensitivity	Specificity	Accuracy	MCC	Coverage	Absolute True	F1 Value
DeepPred-SubMito	-	-	0.79	0.49–0.99	-	-	-
SubMito-XGBoost	-	-	97.7–98.9	-	-	-	-
mRNALoc	62.67–96.28	65.34–99.79	65.19–99.70	0.14–0.95	-	-	-
SCLpred-EMS	-	-	-	0.75–0.86	-	-	-
BUSCA	-	-	-	-	-	-	0.49
SubMitoPred	71.88–85.37	71.19–95.18	71.23–94.37	021–0.68	-	-	-
pLoc-mEuk	-	-	84.34	-	84.34	78.78	-
ERPred	79.84	81.58	81.42	0.42	-	-	-
DeepLoc	-	-	78–92	0.45–0.83	-	-	-
SubNucPred	72.22–86.49	75.44–88.60	75.40–88.89	0.33–0.64	-	-	-
LocTree3	-	-	81-96	-	86–96	-	-
PlantLoc	-	-	80.80	-	-	-	-
iLoc-Hum	79.30	94.99	76.31	-	-	-	-
iLoc-Animal	-	-	-	-	-	0.45	-
iLoc-Plant	-	-	71.70	-	-	-	-
iLoc-Euk	-	-	79.06	-	-	-	-
iLoc-Virus	-	-	78.20	-	-	-	-
iLoc-Gpos	-	-	93.12	-	-	-	-
iLoc-Gneg	-	-	91.40	-	-	-	-
MARSpred	98.33	92.50	96	0.92	-	-	
SCLPred	0.34–0.93	0.39–0.93	-	0.68–0.92	-	-	-
AtSubP	91.00	97.90	-	0.89	-	-	-
Euk-mPLoc 2.0	-	-	64.17	-	-	-	-
PSORTb 3.0	-	-	97.90–98.30	0.79–0.85	-	-	-
YLoc	-	-	48.00–58.00	-	-	-	0.37–0.67
SubChlo	-	-	67.18–89.69	-	-	-	-
MultiLoc2	81.90–89.40	-	77.90–88.90	-	-	-	-
AAIndexLoc	19.00–87.00	29.00–83.00	74.50–77.30	0.23–0.79	-	-	-
Euk-mPLoc	-	-	67.40	-	-	-	-
Hum-mPLoc 3.0	-	-	63.00	-	-	-	0.65
Gpos-PLoc	-	-	82.70	-	-	-	-
Gneg-PLoc	-	-	87.30	-	-	-	-
Virus-PLoc	-	-	80.00–89.20	-	-	-	-
ProLoc-GO	-	-	88.10–88.30	-	-	-	-
ProLoc	-	-	56.37–72.82	-	-	-	-
SherLoc	81.00–94.00	29.00–96.00	85.30–96.20	0.48–0.92	-	-	-
MitPred	56.36	100	93.06	0.72	-	-	-
BaCelLo	-	-	39.40–99.50	-	50.7–97.7	-	-
HSLpred	-	-	84.90	0.80	-	-	-
PSLpred	-	-	86.80–95.20	0.84–0.95	-	-	-
ESLpred	-	-	88.00	0.69–0.91	-	-	-

Note: Performance mentioned here is range of performance for that particular method. '–' represents that method does not used that matrix for the performance.
